# Evaluation of Apically Extruded Debris during Root Canal Retreatment Using ProTaper Next and Reciproc in Severely Curved Canals 

**DOI:** 10.22037/iej.v12i3.15850

**Published:** 2017

**Authors:** Giselle Nevares, Kaline Romeiro, Diana Albuquerque, Felipe Xavier, Howard Fogel, Laila Freire, Rodrigo Cunha

**Affiliations:** a *Department of Operative Dentistry and Endodontics, Dental College of Pernambuco, University of Pernambuco, Camaragibe, PE, Brazil; *; b *Division of Endodontics, College of Dentistry, University of Manitoba, Winnipeg, MB, Canada; *; c * School of Dentistry, University of São Paulo, São Paulo, SP, Brazil*

**Keywords:** Gutta-Percha, Root Canal Filling Materials, Root Canal Retreatment

## Abstract

**Introduction:**

To compare the apical extrusion of debris produced during root obturating material removal from severely curved root canals using either Reciproc (REC) or ProTaper Next (PTN) systems.

**Methods and Materials:**

Twenty-six mesial canals of lower molars were instrumented, filled and allocated into two groups (*n*=13). Micro-computed tomographic images were performed to determine the root canal configuration (Vertucci’s type IV) and initial volume of obturation. One Eppendorf tube was assigned per canal and weighed (10^-4^g) before and after removal of the obturating material. The difference between the initial and final weights was calculated and statistically evaluated.

**Results:**

Apical extrusion of debris was confirmed in all samples, and the mean amount of apical extrusion was similar between both groups (0.061±0.014 g in PTN *vs.* 0.065±0.016 g in REC samples) (*P*<0.05).

**Conclusion:**

Both systems caused apical extrusion of debris with no differences between PTN and REC systems.

## Introduction

Root canal retreatment procedure usually leads to apical extrusion of dentinal debris and root canal obturation material [1]. In addition, irrigants, necrotic pulp tissue remnants, microorganisms and their byproducts may also be pushed to the periradicular tissues [2]. Apical extrusion is undesirable due to its association with post-operative pain and/or edema, being directly related with symptomatic apical periodontitis [3-5] and cytotoxic effects [6]. Thus, efforts must be made to minimize the extrusion of debris trough the apical foramen [4, 5]. 

Although unavoidable, the apical extrusion of debris can be reduced using mechanized removal of obturation material from the root canal [7]. However, this can yield variable results according to the number of instruments, instrument’s design and the kinematics employed [8]. Engine-driven nickel-titanium (NiTi) rotary and reciprocating files for root canal instrumentation have been successfully used for retreatment [9]. ProTaper Next (PTN) system (Dentsply Maillefer, Ballaigues, Switzerland) is rotary system with rectangular cross-section of instruments that must be used with a conventional rotary motion. Due to its offset center of mass and center of rotation, when in motion, the device generates a mechanical wave similar to a sinusoidal wave, making its movement asymmetrical [10]. The system is made of five instruments including X1 (017/0.04), X2 (025/0.06), X3 (30/0.07), X4 (040/0.06) and X5 (050/0.06). The efficacy of PTN system has been tested for the removal of gutta-percha [11] and only one study evaluated apical extrusion of debris produced during this procedure [12] . 

The Reciproc (REC) system (VDW, Munich, Germany) is a single instrument used in reciprocating back-and-forth alternating movements in clockwise and counter-clockwise directions. This single file system is available at three different sizes and tapers; R25 (25/0.08), R40 (40/0.06) and R50 (50/0.05). Those files have an s-shaped cross-section along their active portion, sharp cutting edges and absence of radial lands [13]. Data concerning debris extrusion in retreatment using REC instruments are limited and only available in anterior [2, 14] and premolar [8, 15, 16] teeth. 

This *in vitro* study aimed to compare the apical extrusion of debris produced during obturation material removal from severely curved root canals using REC and PTN instruments. The null hypothesis tested was that there is no difference between the two systems in this regard.

## Materials and Methods

***Sample size calculation***

The sample size was based on a previous study that observed the apical extrusion of debris using mechanized instruments in retreatment [16]. A minimum size of 11 samples per group was required using the test of equal means (*t*-Student; Minitab® Statistical Software 16.1, Minitab Inc., URL: www.minitab.com) with *α*=5%, power of 95% and ratio of 1.00.

***Initial sample selection***

This study was previously revised and approved by the Nevares, de Albuquerque, Freire, Romeiro, Fogel, Dos Santos and Cunha [11] study. Initially, 189 extracted first and second mandibular molars were examined using a stereomicroscope (4× magnification). Only mesial roots with fully formed apices were included. Crowns were adjusted in order to produce samples with standardized lengths of 17 mm. After radiographic examination, teeth with endodontic access, pulp calcification and/or internal resorption were excluded. Angle [17] and radius [18] of curvature were measured using Image J software version 1.46r (National Institutes of Health, Bethesda, MD,USA). The mean angle was 35.5^°^ (standard deviation of 6.86^°^ and coefficient of variation of 19.32%) and the mean radius of curvature was 5.3 mm (standard deviation of 1.73 mm and coefficient of variation of 32.64%). To compare the techniques in the same root, only roots with two separate mesial canals were included. 

After endodontic access, a glide path was created using a #10 K-file. The working length (WL) was set at 1 mm short of the apical foramen. All mesiobuccal and mesiolingual canals were instrumented using the reciprocating WaveOne Small file (21/0.06) (Dentsply Maillefer, Ballaigues, Switzerland). The pulp chamber was irrigated using 2 mL of 2.5% sodium hypochlorite with a 5 mL syringe and a 30 G needle. The file was introduced into the canal until resistance was felt, with 3 in-and-out pecking motions with slight apical pressure. The file was removed and its blades were cleaned using a sponge soaked with alcohol. This procedure was repeated until the file reached the predetermined WL and the irrigation needle reached 2 mm short of the WL. Patency was maintained using a #10 K-file at the apical foramen level. The smear layer was removed using 2 mL of 17% EDTA agitated for 1 min using a sonic device (EndoActivator; Dentsply Tulsa Dental, OK,USA) with a Small tip, followed by 5 mL of 2.5% NaOCl. Canals were dried using paper points. Obturation was performed using a modified hybrid Tagger's technique [19]. The tip of a tapered gutta-percha point (WaveOne Small) was coated with sealer (AH Plus, Dentsply, Tulsa Dental, Tulsa, OK, USA) and placed into the root canal. An engine plugger was placed 4 to 5 mm into the canal for thermo mechanical compaction. The pulp chambers were sealed using temporary restorative material, stored at 37^º^C with 100% relative humidity for 30 days. The teeth were radiographed buccolingually and mesiodistally to assess the quality of the obturation. 

***Sample selection using Micro-CT scanning***

Teeth were scanned to confirm the selection of type IV Vertucci canal configuration [20] and mean initial volume of obturation. A SkyScan 1176 micro-CT scanner (Bruker-microCT, Kontich, Belgium) was used, which allows for scanning of high-density objects, and images were reconstructed with NRecon v.1.6.9 software (Bruker-microCT) using the modified Feldkamp cone-beam reconstruction algorithm (scanning: 90 kV, 258 µA, 360^°^ rotation, 0.5^°^ rotation step, 17.42 μm voxel size). Preoperative volumes of the obturation material in the mesiobuccal and mesiolingual canals were measured in cubic millimeters for the entire canal and separated by thirds (cervical, middle, and apical). A total of 13 roots were selected for the final sample.

***Initial weighting of Eppendorf tubes***

**Figure 1:**
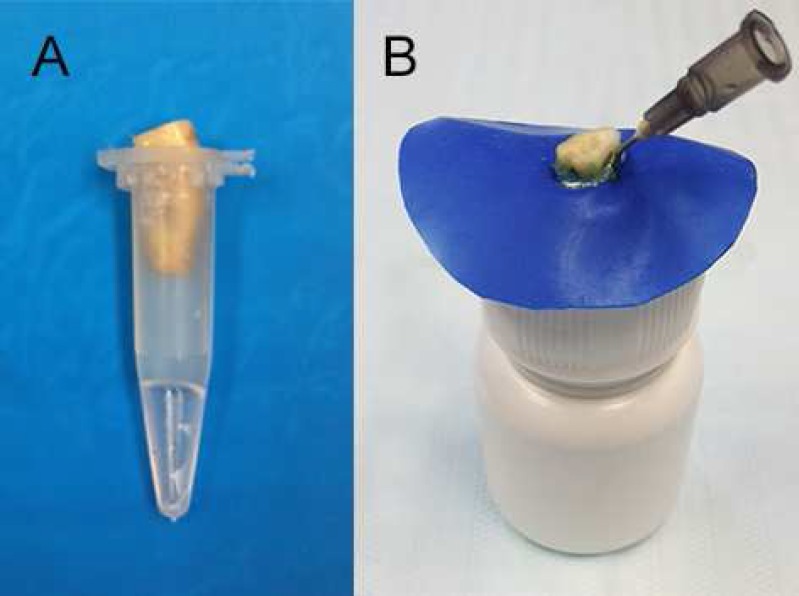
)* Eppendorf tube; *B)* Modified apparatus used to evaluate the apical extrusion of debris*

The experimental model described by Myers and Montgomery [21] with previously suggested modifications [22] was used to collect the debris and evaluate debris extrusion (Figure 1). One Eppendorf tube was assigned for each mesial canal. An opening was created on each Eppendorf tube cap, according to each root's anatomical configuration and the roots were affixed with cyanoacrylate to prevent unintentional leakage of irrigating solution. Each tube was numbered and individually weighed on an analytical balance (accuracy of 10^-4 ^g). Five consecutive weightings were conducted for each tube, and the highest and lowest values were discarded. The arithmetic mean of three weights obtained was regarded as the initial weight of the Eppendorf tube. A 27G needle was folded and inserted into the Eppendorf cap to balance internal and external pressure. The Eppendorf tubes were stored in an opaque container, covered with a rubber sheet to avoid visualization by the operator during instrumentation. 

***Removal of obturation material***

The experimental groups were defined according to the system chosen for the obturation material removal. A total of 26 paired canals were randomized (www.random.org), resulting in an equal number of mesiobuccal and mesiolingual canals in each group (*n*=13), and both systems were tested on the same root. The removal of obturation material was considered when no gutta-percha or sealer was visible between the cutting blades with the aid of a dental operating microscope (8× magnification). A stainless steel file #10 was used to provide patency in both groups.

**REC Group**: The R25 file (25/0.08) was used in reciprocating motion and the technique for obturation material removal and irrigation was similar to that used in the initial instrumentation. The file was used until the WL was reached. The total volume of 2.5% NaOCl was 20 mL and the final irrigation using EDTA was not performed.

**PTN Group: **The X3 file (30/0.07) was used in the cervical and middle thirds and X2 file (25/0.06) in the apical third in continuous rotary motion. The engine motor was set for 500 rpm and 3 N.cm of torque. At every 3 backward and forward movements, the file was removed and cleaned using a sponge soaked with alcohol. The files were used until they reached the WL measurements. Irrigation was conducted using the same protocol and volume as for the obturation material removal on the REC group.

***Final weighing of Eppendorf tubes***

The teeth were removed from the Eppendorf tubes, and their roots were washed with 1 mL of 2.5% NaOCl to collect the debris that had adhered to their outer side. All tubes were incubated at 37˚C for 15 days to allow the evaporation of the remaining irrigant from the tubes [22] . After the incubation period, a final weighting was performed in the same manner as the initial weighting. 

***Statistical analyses***

Before retreatment procedures, for comparisons of the mean initial volume of the canal obturation *t* test with equal variances was used. 

Verification of the hypothesis of equality of variances was performed using Levene's *F* test. The margin of error used in the statistical tests was 5.0%. 

The differences between initial and final mean weights were calculated and statistically evaluated using Wilcoxon's test for paired data for the intra-groups and Mann-Whitney's test for inter-groups comparisons. In all the statistical analyses, SPSS 21 (IBM SPSS Inc, Chicago, IL, USA) software was used.

## Results

The mean initial volume (in mm^3^) of the canal obturation in the total (3.930 ± 0.850 PTN *vs*. 3.970 ± 1.130 REC), cervical (2.304 ± 0.636 PTN *vs*. 2.328 ± 0.759 REC), middle (1.289 ± 0.304 PTN *vs*. 1.456 ± 0.473 REC) and apical (0.339 ± 0.135 PTN *vs*. 0.387 ± 0.187 REC) thirds was similar between the samples (*P*>0.05).

Table 1 presents the data regarding the debris extrusion per group. Both instrumentation systems produced apically extruded debris in all samples. No significant differences were found between the REC and PTN groups (*P*>0.05). The data variability was small in all the analysis, as shown by the coefficient of variation in Table 1. 

**Table 1:** Mean (SD) and coefficient of variation (CV) of the amounts of debris extruded apically during obturation material removal (accuracy of 10^-4 ^grams) (*n*=13

**Group (N)**	**Statistics**	**Evaluation**		**Difference between evaluations**
		**Initial**	**Final**	
**PTN**	**Mean (SD)**	0.691 (0.053)	0.752 (0.057)	0.061 (0.014)
	**CV (%)**	7.670	7.580	22.951
**REC**	**Mean (SD)**	0.691 (0.062)	0.756 (0.064)	0.065 (0.016)
	**CV (%)**	8.973	8.466	24.615
	***P*** **-value**	0.880	0.840	0.201

## Discussion

In the present study, effort was placed into balancing the samples to minimize the influence of canal anatomy. The distribution of the groups was similar with respect to angle and radius of canal curvature and was classified as severe, as in previous studies [17, 23]. Furthermore, the initial obturation volumes were similar between the groups (*P*>0.05). Also, the use of micro-CT scanning in this study allowed for the visualization of anatomical characteristics. The mesial roots selected were in accordance with type IV Vertucci canal configuration [20]. This was the same as the study by Gergi *et al.* [24] in which roots had two canals that were separate and distinct from the pulp chamber to the apex. Given that the mesiobuccal has a tendency for sharper curvatures in comparison to the mesiolingual canal [25], an equal distribution between both groups was assured. No significant difference between the REC and PTN systems was found with respect to the amount of apically extruded debris. Therefore, the null hypothesis was accepted. The instrument design and kinematics is different in both systems. The last apical file has a #25 tip diameter in both systems but the instruments are different with regard to taper and file design. R25 file has an initial 8% taper in its first 3 mm decreasing from this point to D16 [13]. According to the manufacturer, the X2 file has a 6% taper over the initial 3 mm followed by an increasing and decreasing percentage tapered design varying between 4% and 7% to D16. Yilmaz and Ozyurek [12] compared the amount of debris extruded from the apex during retreatment procedures with PTN and REC systems. Although the instruments X5 (50/0.06) and R50 (50/0.05) were added to apical enlargement in PTN and REC groups, respectively. In this previous study, the REC group extruded significantly more debris than the PTN group. Instruments with greater taper could produce more dentin debris, thus the amount of apically extruded debris would be increased [13]. However, this finding was not seen in both studies. 

When comparing both systems used in this study, a previous study showed that R25 and X2 files have a similar cutting ability [23]. A factor that seems to influence the cutting ability of PTN systems is the kinematics [26]. This system allows a larger envelope of motion when compared to files with centralized mass and rotation axis [26]. Notwithstanding, Caviedes-Bucheli *et al. *[3] noticed that the instrument’s design is the most influential factor on the extrusion of debris, regardless of the kinematics used. The cutting efficiency of REC system has been more frequently ascribed to its cross-section than to the reciprocating motion [26]. In spite of the systems dissimilarities regarding the cross-sections, they both have 2 cutting edges that are in contact with the root canal walls. This may also contribute to the similar results between the two systems studied. 

Regarding the number of instruments, in the present study the REC group used only 1 instrument for the obturation material removal, whereas the PTN group used 2 instruments. Kasikci Bilgi *et al.* [27] study compared the amount of apically extruded debris after using Reciproc (R25 and R40 (40/.06) instruments) and ProTaper Universal Retreatment D1 (30/.09), D2 (25/.08) and D3 (20/.07) instruments (Dentsply Tulsa Dental Specialties) followed by the use of supplementary X2, X3 and X4 (40/.06) instruments from ProTaper Next system. The debris extruded was not statistically significant between the groups. Previous studies reported that the high number of instruments used might be another factor that accounts for the greater amount of debris extrusion [8]. The number of instruments did not seem to influence the results by Kasikci Bilgi *et al.* [27] and our studies. 

Part of the debris produced is removed during the root canal irrigation and aspiration procedures [28]. In this study, all samples were irrigated with the same technique and the volume was standardized. In order to better simulate the clinical procedure, NaOCl was used due to its well-established status in similar methodology for the extrusion of debris [8]. Solvent was not used in the current study to eliminate the chemical melting of gutta-percha and the adherence of a thin layer of this material to the canal walls [29]. In addition, softened gutta-percha may be pushed into irregularities, hindering the cleaning process [30]. 

To collect the apically extruded debris, this study relied on Myers and Montgomery [21] work and it is accepted as a well-established* in vitro* methodology [5, 22]. Clinically, periapical tissues could act as a physical barrier to the apical extrusion of debris [31]. However, the apical extrusion of debris following the use of motor-driven instruments does occur clinically and is directly related to apical periodontitis and to periodontal ligament inflammation [3]. No attempts were made to simulate periapical tissues in this study or in the original methodology [21]. Apical barriers using agarose gel [1] and floral foam [32] have been used. However, according to Mitchell, Yang and Baumgartner [33], the agarose gel density does not simulate intact periapical tissues or periradicular lesion conditions. In addition, the sponge may absorb the irrigant and the extruded debris, altering its quantification [34]. 

## Conclusion

Under the conditions of the present study, when REC and PTN were used to remove obturation material both systems produced apically extruded debris and there was no difference in the amount of apically extruded debris between both groups.
